# Navigating the Labial Artery: A Safer Approach to Submucosal Lip Filler Techniques

**DOI:** 10.3390/life15040509

**Published:** 2025-03-21

**Authors:** Bruna S. F. Bravo, Leonardo G. Bravo, Beatriz G. Cornachini, Mariana C. Elias, Gabriel L. T. Alves

**Affiliations:** Department of Dermatology, Bravo Private Clinic, Avenida Ataulfo de Paiva 245 Quinto Andar Leblon, Rio de Janeiro 20440-032, Brazil

**Keywords:** lip augmentation, submucosal layer, labial artery, ultrasound, hyaluronic acid

## Abstract

Lip augmentation using hyaluronic acid (HA) fillers has become one of the most popular non-surgical aesthetic procedures, yet it is not without risks, particularly vascular complications. This study explores a novel vertical injection technique for submucosal placement of HA fillers, designed to enhance safety and achieve natural-looking outcomes. Ten patients underwent lip augmentation using the proposed technique, which involved ultrasound-guided injections at predefined entry points to target intralabial compartments while minimizing the risk of labial artery injury. The results demonstrated high patient satisfaction, with significant improvements in lip volume and contour. The submucosal placement yielded subtle, natural results with fewer visible irregularities compared to superficial injections. No major complications, such as vascular occlusion, were observed, and minor side effects, including transient swelling and redness, resolved within 48 h. The use of ultrasound imaging allowed precise filler placement and reduced the risks associated with traditional horizontal injection paths parallel to the labial arteries. While the findings highlight the technique’s potential in terms of safety and efficacy, limitations include the small sample size, short-term follow-up, and the absence of a control group. Further studies with larger cohorts and comparative analyses are needed to validate long-term outcomes. This innovative approach underscores the importance of anatomical precision and advanced imaging technologies in enhancing safety and aesthetic outcomes in lip augmentation.

## 1. Introduction

Over the last decade, lip augmentation using hyaluronic acid (HA) fillers has increased in popularity, becoming one of the most sought-after non-surgical aesthetic procedures. According to the American Society of Plastic Surgeons (ASPS), the number of lip augmentation procedures with injectable materials reached an all-time peak with 1,439,291 procedures performed in 2023, reflecting a growing desire for enhanced lip volume and contour, and thereby underlining the aesthetic importance of this facial region [1,2]. As the demand for lip augmentation continues to grow, so does the imperative for injectors to optimize patient safety.

In fact, despite the widespread use and efficacy of HA fillers for lip enhancement, the procedure is not without risks [3,4]. One of the primary concerns associated with lip augmentation is the potential for vascular complications, such as inadvertent injection into or compression of the labial artery [5]. These complications might culminate in severe adverse events, including tissue necrosis and, in rare cases, blindness [6,7]. Therefore, a thorough understanding of the lip’s complex anatomy is crucial for practitioners to perform not only effective but also safe lip augmentations [8].

The anatomy of the lips is complex and multi-layered, comprising subcutaneous, intramuscular, and submucosal planes, each with distinct characteristics and functions [9]. The subcutaneous layer contains fat and connective tissue, providing the lips with their shape and volume. Beneath this layer lies the orbicularis oris muscle, which plays a crucial role in lip movement and expression. The deepest layer, the submucosal layer, is situated just above the mucosal lining of the oral vestibulum and is significant for its vascular content, particularly the superior and inferior labial artery in the upper and lower lip, respectively.

The labial artery (LA), a vital branch of the facial artery, is primarily responsible for the arterial supply of the lips. A thorough understanding of its topographic course is critical for any procedure involving lip injections. The LA bifurcates into the superior labial artery (SLA) and the inferior labial artery (ILA), which enter the lips at the oral commissures [9,10]. Typically, the SLA traverses the upper lip, while the ILA supplies the lower lip. These arteries follow a tortuous path, often located within the submucosal layer, making their precise localization challenging [11]. The SLA usually enters the lip within 0.5 cm of the oral commissure, traveling medially and often being situated more deeply compared to the ILA, which is generally more superficial and smaller in diameter at the midline. Anatomical studies have shown that the labial artery’s location can vary, with it being found in the submucosal layer in approximately 58.5% of cases, the intramuscular layer in 36.2%, and the subcutaneous layer in 5.3% [9].

**Figure 1:**
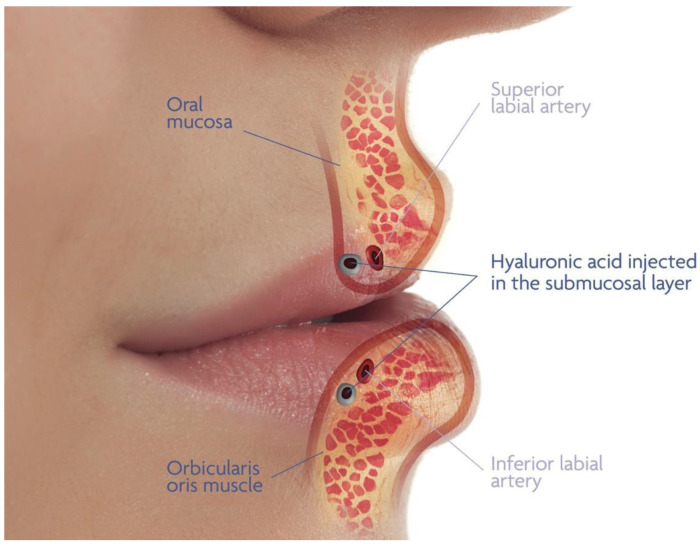
Graphical illustration of the innovative lip-filling technique presented in this study.

Understanding the detailed course of the labial artery and the layered anatomy of the lips is essential for minimizing risks and achieving optimal results in lip augmentation procedures. This study aims to explore a novel submucosal injection technique for HA fillers, designed to enhance safety by strategically navigating the lip’s anatomy and avoiding the labial artery (Figure 1).

## 2. Materials and Methods

### 2.1. Study Setup

This prospective case study included ten patients who underwent lip augmentation using HA fillers. The study was conducted between June and July of 2024 at the private practice of the senior author, Bravo Clinic in Rio de Janeiro, Brazil. All patients were selected based on their desire for natural-looking lip enhancement and their consent to participate in a study focusing on submucosal injection techniques. Exclusion criteria included previous lip surgery, active infections, or known hypersensitivity to HA fillers. Patient satisfaction was assessed during follow-up consultations through structured discussions. Patients were asked to describe their perceived aesthetic outcome, specifically regarding lip volume and contour, and whether they would choose to undergo the procedure again.

### 2.2. Procedure

The HA filler used was Rennova^®^ Lips Plus (Rennova, Goiana, Brazil), a high-density product suitable for deep tissue augmentation. The procedures were performed in a sterile environment, and all patients received a topical anesthetic to minimize discomfort.

**Figure 2:**
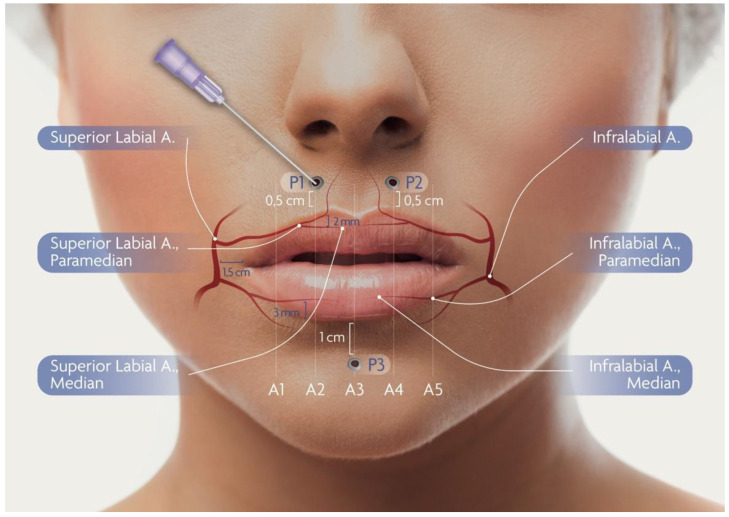
Overview of the five anatomical reference points (A1: right oral commissure, A2: lateral border of the right nasal wing, A3: subnasale, A4: lateral border of the left nasal wing, A5: left oral commissure) and three entry points used for filler injection (P1: 0.5 cm above the vermilion border, in line with the lateral border of the right nasal wing (A2); P2: 0.5 cm above the vermilion border, in line with the lateral border of the left nasal wing (A4); P3: 1 cm below the vermilion border, in line with the subnasale (A3)).

**Figure 3:**
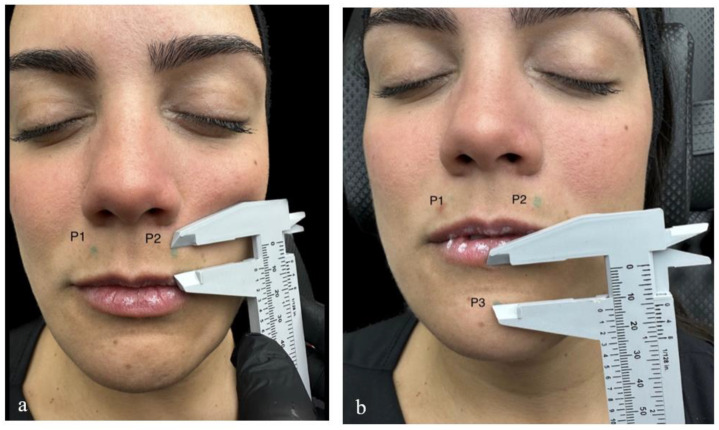
Patient image illustrating the three entry points, P1–P3, used for filler injection. P1: 0.5 cm above the vermilion border, in line with the lateral border of the right nasal wing (A2); P2: 0.5 cm above the vermilion border, in line with the lateral border of the left nasal wing (A4); P3: 1 cm below the vermilion border, in line with the subnasale (A3)).

**Figure 4:**
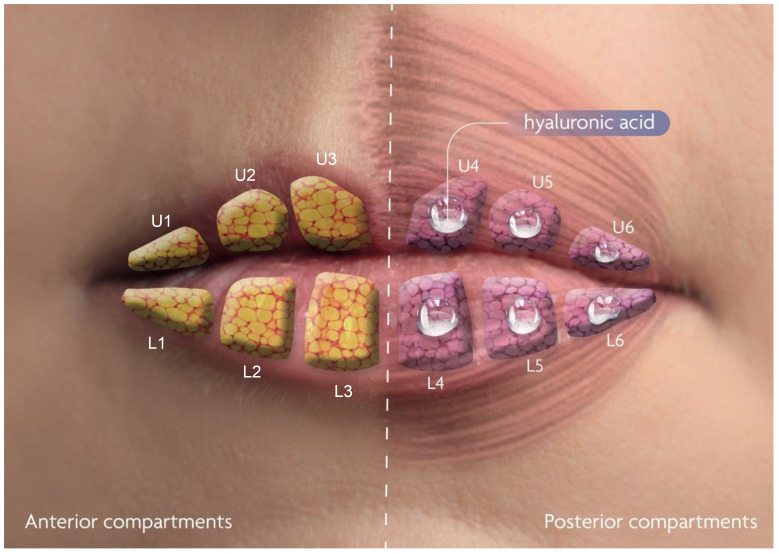
Overview of the intralabial compartments as defined by Cotofana et al. [8]. Hyaluronic acid (HA) was injected into the posterior compartments of both the upper and lower lips. Our technique involved six injection sites in the upper lip: U1–U3 correspond to the right posterior upper-lip compartments, while U4–U6 correspond to the left posterior upper-lip compartments as shown in this figure. Likewise, six injection sites were used in the lower lip: L1–L3 correspond to the right posterior lower-lip compartments, and L4–L6 correspond to the left posterior lower-lip compartments as shown in this figure.

For the product administration, 3 entry points were utilized (Figure 2 and Figure 3; P1: 0.5 cm above the vermilion border, in line with the lateral border of the right nasal wing; P2: 0.5 cm above the vermilion border, in line with the lateral border of the left nasal wing; P3: 1 cm below the vermilion border, in line with the subnasale). HA was injected through entry points P1 and P2 into six sites in the upper lip (U1-6), and through entry point P3 into six sites in the lower lip (L1-6). U1-6 and L1-6 correspond to the posterior intralabial upper and lower lip compartments defined by Cotofana et al., respectively (Figure 4; U1: right lateral posterior upper-lip compartment, U2: right middle posterior upper-lip compartment, U3: right medial posterior upper-lip compartment, U4: left medial posterior upper-lip compartment, U5: left middle posterior upper-lip compartment, U6: left lateral posterior upper-lip compartment; L1: right lateral posterior lower-lip compartment, L2: right middle posterior lower-lip compartment, L3: right medial posterior lower-lip compartment, L4: left medial posterior lower-lip compartment, L5: left middle posterior lower-lip compartment, and L6: left lateral posterior lower-lip compartment) [8].

Key steps include asepsis of the entire lip area with chlorhexidine alcohol, infiltrative anesthesia of both the right and left infraorbital nerves with 1 mL of injectable lidocaine 2% (20 mg/mL) on each nerve, and infiltrative anesthesia of both the right and left mental nerves with 0.5 mL of injectable lidocaine 2% (20 mg/mL). Injectable lidocaine 2% + epinephrine was used to anesthetize the entry points P1, P2, and P3 (0.2 mL/entry point). Entry points P1 and P2 in the cutaneous part of the upper lip were used to insert the 22G 40 mm cannula into the upper lip, and entry point P3 in the cutaneous part of the lower lip was used to insert the 22G 40 mm cannula into the lower lip. Injection volumes were defined based on the volume of the intralabial compartments as calculated by Cotofana et al. [8] (U1: 0.05 mL, U2: 0.05 mL, U3: 0.1 ml, U4: 0.1 mL, U5: 0.05 mL, U6: 0.05 mL, and L1: 0.05 mL, L2: 0.1 mL, L3: 0.15 mL, L4: 0.15 mL, L5: 0.1 mL, and L6: 0.05 mL). All of the HA were placed in the submucosal layer, below the wet–dry lip junction and posterior to the SLA and ILA, as confirmed by ultrasound imaging. A pressured massage was performed on the lips at the end of the procedure for final accommodation of the HA. A step-by-step video demonstrating the injection technique is provided in Supplementary File S1.

### 2.3. Ultrasound Analysis

**Figure 5:**
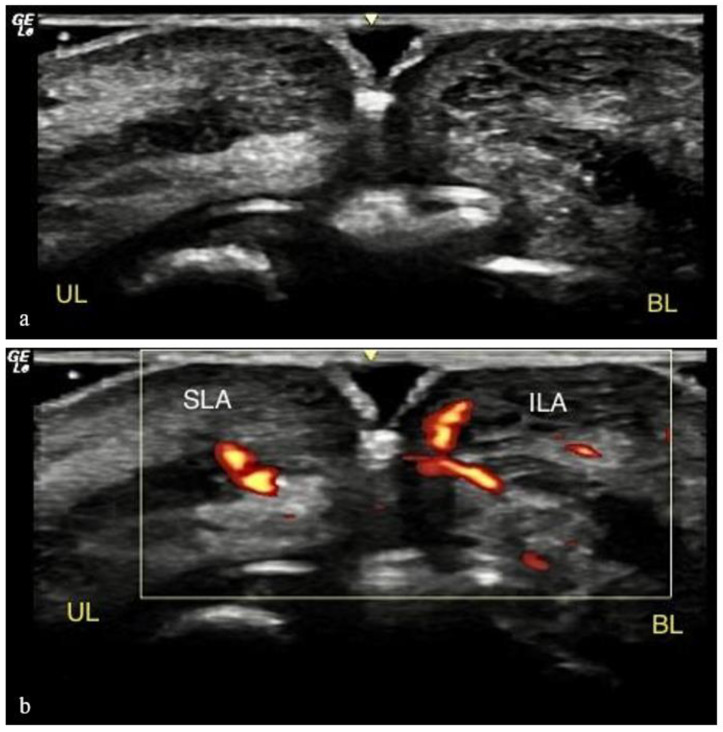
Pre-procedure imaging (T0) involved placing the probe vertically along the five reference lines (A1–5) to capture baseline images in B mode (**a**) and Power Doppler Imaging (PDI) mode (**b**). The ultrasound equipment used included the Logic GE NextGen Ultrasound (Elk Grove, CA, USA, 2023) combined with transparent conductor gel for ultrasound (RMC, Amparo, Brazil, 2024) and a GE L8-18i-SC 8–18 MHz “Hockeystick” probe.

**Figure 6:**
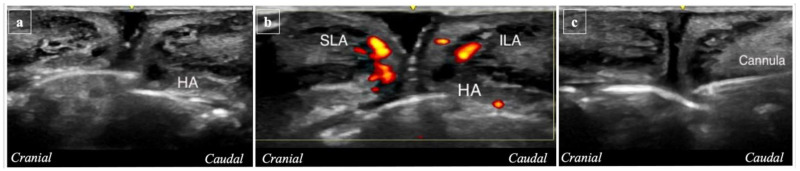
During the procedure (T1), the ultrasound probe was placed on L5 while the 22G 40 mm cannula was inserted at P3 and evaluated in B mode (**a**) and Power Doppler Imaging (PDI) mode (**b**). In addition, the probe was placed on A3 while the 22G 40 mm cannula was inserted at P2 (**c**).

**Figure 7:**
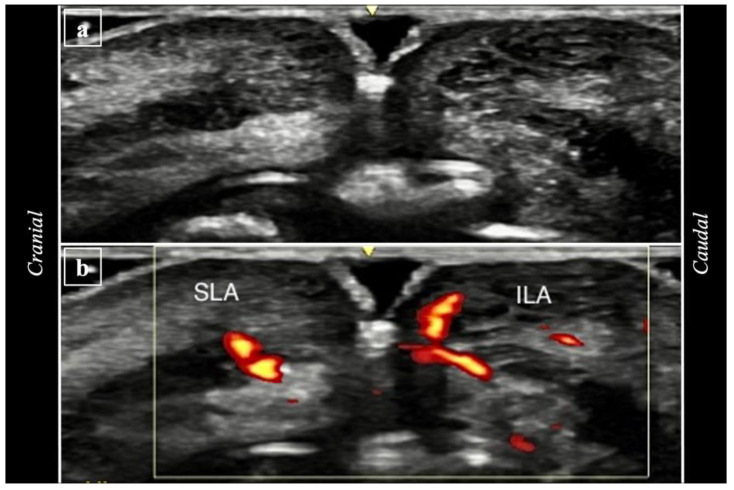
Example of imaging after the procedure (T2), taken with the ultrasound probe being placed on A3.

Reference points for ultrasound measurements were defined as 5 vertical lines (Figure 2; A1: right oral commissure, A2: lateral border of right nasal wing, A3: subnasale, A4: lateral border of left nasal wing, A5: left oral commissure). Ultrasound analyses were performed at three time points:

(i) Pre-procedure imaging (T0) involved placing the probe vertically along the five reference lines to capture baseline images in B mode (Figure 5a) and Power Doppler Imaging (PDI) mode (Figure 5b).
(ii) During the procedure (T1), imaging involved placing the probe on L5 while the 22G 40 mm cannula was inserted in P3 (Figure 6a,b) and on A3 while the 22G 40 mm cannula was inserted in P2 (Figure 6c).
(iii) Post-procedure imaging (T2) involved capturing new images in the same vertical positions in A1, A2, A3, A4, and A5 (Figure 7).

## 3. Results

All ten patients included in this study were female. The mean age was 34.6 ± 4.7 years, ranging from 25 to 45 years. Regarding previous exposure to lip fillers, all patients had either never received HA injections or had undergone their last HA lip augmentation at least one year prior to the study. None of the participants had permanent filler materials in their lips. All patients expressed high satisfaction with the aesthetic outcomes, highlighting natural-looking results and noticeable improvements in lip volume and contour. Additionally, all ten patients stated that they would undergo the procedure again.

**Figure 8:**
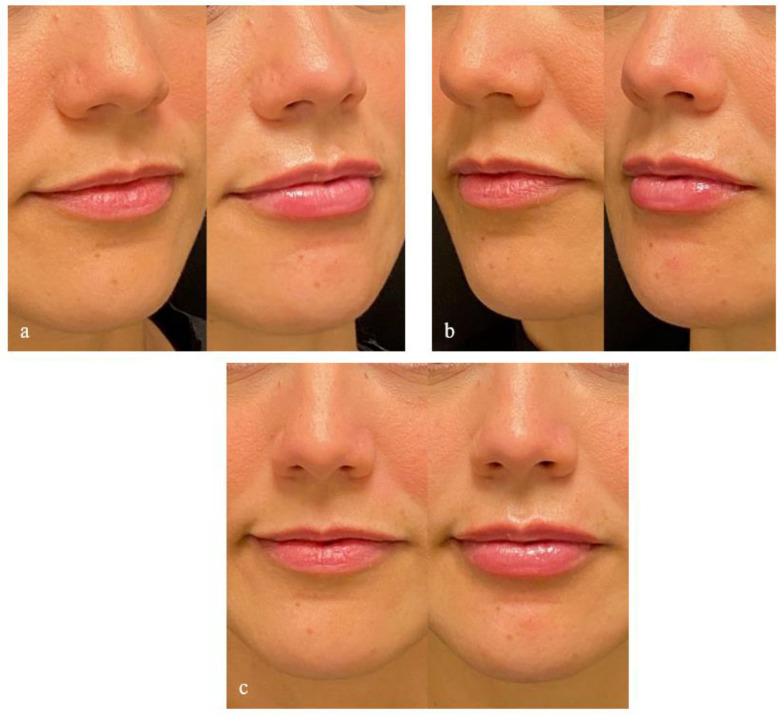
(**Left**) Pre-procedure image showing the patient’s disproportionate upper lip volume compared to the lower lip. (**Right**) Post-procedure photograph following enhancement of lip volume. (**a**) Right oblique view; (**b**) left oblique view; (**c**) anterior view.

The submucosal placement of the HA filler resulted in a more natural and delicate appearance compared to superficial injections (Figure 8). The deeper placement of the filler resulted in a more subtle outcome both upon inspection and palpation. No major complications such as vascular occlusion or ischemia were observed. Minor side effects included transient swelling and redness, which resolved within 48 h. Post-procedure discomfort was minimal, with patients resuming normal activities the same day.

## 4. Discussion

Our ten-patient case study highlights the potential of a vertical injection technique for submucosal HA filler placement as an innovative method for lip augmentation. This approach showcases how the use of ultrasound guidance can foster safety and efficacy, even in layers that are typically deemed to be at greater risk of vascular injury [8].

When evaluating an injection technique—regardless of its clinical objective and the facial region targeted—two factors are pivotal to consider: safety and efficacy [12]. Speaking about safety, the topographical anatomy of vessels in this facial region is crucial. Despite the cannula’s proximity to the labial arteries in the upper and lower lip, the distinct characteristic of the proposed injection technique is its vertical approach: by using midline entry points and advancing the cannula perpendicularly to the labial artery [13,14,15], this method minimizes the risk of vascular complications. Traditional horizontal injection paths parallel to the labial artery follow the course of the artery for a longer distance [14] and, therefore, increase the likelihood of encountering the artery. In contrast, the vertical path ensures that the filler is placed below the wet–dry lip junction, behind the artery within the submucosal plane, reducing the risk of occlusion or ischemia. Safe and precise product placement can be achieved by the choice of administration device (i.e., 22G 40 mm cannula) and imaging modality (i.e., ultrasound imaging) [16]. Notably, ultrasound-guided imaging has been reported to aid the injector in the precise placement of soft tissue fillers in a plethora of previous publications [17,18,19,20]. Patients reported high satisfaction with the natural and delicate appearance of their augmented lips. In fact, the deeper placement of the filler allows for a more subtle enhancement, with the filler being less visible and detectable. This submucosal placement provides a smoother contour and prevents the over-projection and irregularities often seen with superficial injections—an effect that can be attributed to the surplus of soft tissue covering the product when compared to more superficial injection techniques. The use of a blunt-tip cannula contributes to patient comfort by reducing tissue trauma during the injection process [21]. Patients experienced minimal discomfort during and after the procedure, with transient swelling and redness resolving quickly. The integration of ultrasonography (US) provided real-time visualization of the lip’s vascular anatomy, allowing for precise filler placement while avoiding critical structures. This technology not only enhanced safety but also offered valuable insights into the dynamics of filler distribution within the submucosal layer.

In general, US has become an indispensable tool in aesthetic medicine, significantly improving both safety and efficacy in cosmetic procedures. Its application is particularly crucial for submucosal and deep dermal injections, where inadvertent vascular compromise may lead to severe complications, including tissue necrosis and, in rare cases, blindness [22]. By offering detailed vascular mapping, US enables practitioners to identify and avoid blood vessels, thus minimizing the risk of intravascular filler placement. In addition, it allows for a more comprehensive assessment of individual anatomical variations, enhancing procedural accuracy and facilitating individualized treatment plans tailored to a patient’s unique anatomy [22]. Beyond pre-procedural planning, US plays a pivotal role in the prompt detection and management of filler-related adverse events [23]. It allows clinicians to promptly identify signs of vascular compromise, filler migration, inflammatory reactions, and granuloma formation [23]. In cases of vascular occlusion, US-guided hyaluronidase administration ensures the targeted enzymatic degradation of HA fillers, facilitating faster and more effective resolution of complications while preserving surrounding tissue integrity [22,23]. Of note, US also provides a reliable method for distinguishing different filler materials based on their echogenic properties—an essential capability when treating patients with unclear injection histories or those who have had previous treatments performed by other providers [24]. As the field of aesthetic medicine continues to evolve, US is emerging as a cornerstone of safe and effective injectable treatments [18]. Its routine integration into clinical cosmetic practice represents a paradigm shift toward more predictable, evidence-based outcomes—ultimately enhancing patient satisfaction while minimizing procedural risks.

### Limitations

The small sample size and short-term follow-up limit the study’s generalizability and the ability to assess long-term outcomes. Larger controlled trials with extended follow-up periods are necessary to validate the technique’s effectiveness and durability. The lack of a control group also prevents a direct comparison with other established techniques, highlighting the need for comparative studies. The technique’s dependency on practitioner skill and experience is another limitation. Similarly, another limitation of this study is the variability in ultrasound proficiency among operators. Ultrasound utilization requires specialized training and interpretation skills, and differences in expertise may contribute to variations in results. Consistent practice and standardized training protocols are essential to improve reliability and reproducibility across different clinical settings.

## 5. Conclusions

The vertical injection technique for deep submucosal HA filler placement represents a promising alternative to traditional techniques in lip augmentation, offering a relatively safe and effective method of achieving natural and aesthetically pleasing results. By respecting the anatomical course of the labial artery and adopting a perpendicular injection path, practitioners can minimize the risk of vascular complications while enhancing patient satisfaction.

## Data Availability

Data are contained within the article.

## Supplementary Materials

The following supporting information can be downloaded at: https://www.mdpi.com/article/10.3390/life15040509/s1.
